# Graft Preservation Solution DuraGraft^®^ Alleviates Vascular Dysfunction Following In Vitro Ischemia/Reperfusion Injury in Rats

**DOI:** 10.3390/ph14101028

**Published:** 2021-10-09

**Authors:** Sevil Korkmaz-Icöz, Belinda Ballikaya, Jasmin Soethoff, Patricia Kraft, Alex Ali Sayour, Tamás Radovits, Sivakkanan Loganathan, Matthias Karck, Gábor Szabó, Gábor Veres

**Affiliations:** 1 Department of Cardiac Surgery, University Hospital Heidelberg, 69120 Heidelberg, Germany; belinda.ballikaya@hotmail.com (B.B.); jasmin.soethoff@med.uni-heidelberg.de (J.S.); kraft@uni-heidelberg.de (P.K.); alexali.sayour@gmail.com (A.A.S.); sivakkanan@gmail.com (S.L.); matthias.karck@med.uni-heidelberg.de (M.K.); Gabor.Szabo@uk-halle.de (G.S.); Gabor.Veres@uk-halle.de (G.V.); 2 Heart and Vascular Center, Semmelweis University, 1122 Budapest, Hungary; radovitstamas@yahoo.com; 3 Department of Cardiac Surgery, University Hospital Halle (Saale), 06120 Halle, Germany

**Keywords:** ischemia/reperfusion, endothelial function, DuraGraft^®^, coronary artery bypass grafting

## Abstract

Vascular ischemia/reperfusion injury (IRI) in patients undergoing coronary artery bypass grafting can result in graft failure and the need for repeat revascularization procedures. DuraGraft^®^ has been shown to protect structure and function in saphenous vein grafts against IRI. We compared the effect of DuraGraft^®^ to saline solution on arterial grafts submitted to IRI. Rat thoracic aortic rings were harvested and immediately mounted in organ bath chambers (control, *n* = 7 rats) or underwent cold ischemic preservation either in saline (IR, *n* = 9 rats) or DuraGraft^®^ (IR+Dura, *n* = 9 rats). Vascular function was measured ex vivo and immunohistochemistry was performed. Impaired maximum vasorelaxation (R_max_) to ACh in the IR-group compared to controls was ameliorated by DuraGraft^®^, indicating an improvement in endothelial function (R_max_ to ACh (%): IR + Dura 73 ± 2 vs. IR 48 ± 3, *p* < 0.05). Additionally, decreased aortic ring sensitivity to ACh (pD_2_-value: -log 50% maximum response) seen after IR in the saline group was increased by DuraGraft^®^ (pD_2_ to ACh: IR+Dura 7.1 ± 0.1 vs. IR 6.3 ± 0.2, *p* < 0.05). Impaired maximum contractile response to phenylephrine and high potassium chloride concentrations in the IR group compared to controls was significantly improved by DuraGraft^®^. DuraGraft^®^ alleviates vascular dysfunction following IRI by reducing nitro-oxidative stress and the expression of ICAM-1, without leukocytes engagement.

## 1. Introduction

Coronary artery bypass grafting (CABG) with autologous conduits is a common surgical operation to redirect blood flow to the ischemic myocardium [1]. Ischemia/reperfusion (IR) injury (IRI) occurs during graft harvest and implantation and can lead to endothelial dysfunction. Reperfusion of ischemic tissue results in injury that is manifested by rapid restoration of a physiologic pH, calcium overload, cellular ATP degradation, neutrophil influx, formation of toxic reactive oxygen species (ROS), release of pro-inflammatory cytokines and enhanced endothelial expression of adhesion molecules [2]. These events are key mediators of bypass graft failure compromising long-term clinical outcomes [3]. To date, prior CABG, saphenous vein grafts (SVGs) and free arterial grafts are typically stored in physiological saline solution or autologous whole blood, which are not sufficiently able to protect the endothelium from IRI and prevent graft disease and failure [4]. Therefore, there is an urgent necessity for improved graft preservation solutions [5].

DuraGraft^®^, the only clinically approved vascular preservation solution, is recognized to protect saphenous vein grafts (SVG), the most often used conduits, during the ischemic interval [6]. This one-time intraoperative treatment protects the integrity and function of the endothelium from IRI. It is formulated into a pH-buffered and ionically balanced physiological solution, containing salts, antioxidants, L-glutathione, L-ascorbic acid, L-arginine (a substrate for nitric oxide synthase in endothelial cells) and other additives. A retrospective analysis has demonstrated that intraoperative treatment of SVGs with Duragraft^®^ significantly reduced clinical post-CABG complications [7]. It has been also shown that DuraGraft^®^ preserves structural viability and integrity of human saphenous vein segments and isolated pig mammary veins during ischemia, whereas saline blood and even buffered solutions do not [8]. 

Although several studies have examined the protective effect of DuraGraft^®^ on vein grafts, experimental data describing its effect on arterial grafts are limited. In the present study, we therefore investigated the impact of DuraGraft^®^ against saline, a recognized storage solution, on arterial grafts in a rat model of in vitro vascular IRI.

## 2. Results

### 2.1. Effect of DuraGraft^®^ on Aortic Vasoreactivity Following Vascular IRI

#### 2.1.1. Effects of DuraGraft^®^ on Contractility

**Figure 1:**
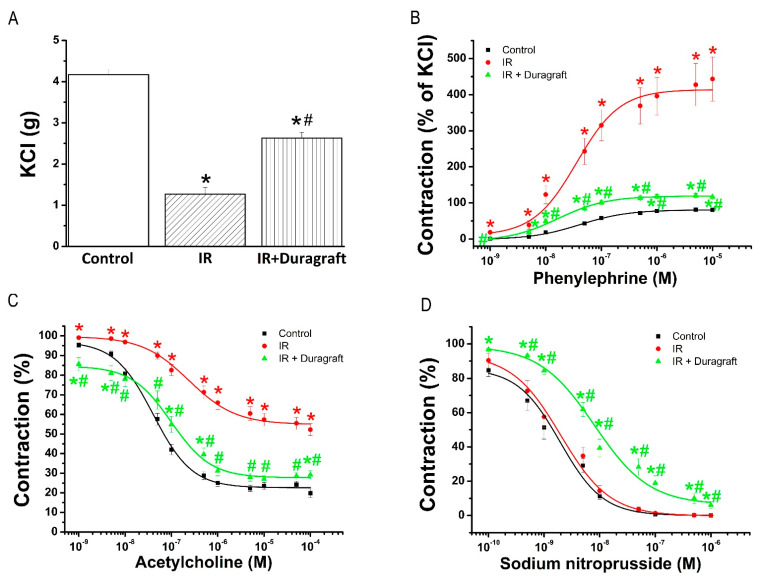
Effect of DuraGraft^®^ on vascular contractile and relaxation responses after ischemia/reperfusion (IR) injury. Contractile responses (**A**) to high K^+^-induced depolarization and (**B**) phenylephrine (expressed as percentage of the maximum contraction induced by 80 mM potassium chloride (KCl), and (**C**) acetylcholine-induced endothelium-dependent- and (**D**) sodium nitroprusside-induced endothelium-independent vasorelaxation of isolated aortic rings. Results are represented as mean ± SEM. * *p* < 0.05 versus control; # *p* < 0.05 versus IR. *n* = 21–36 aortic rings from 7–9 rats/group.

To investigate the effect of DuraGraft^®^ on vascular contractile response after IRI, aortic rings were subjected to ex vivo high potassium- or phenylephrine-induced contraction. Decreased depolarization-induced contraction of smooth muscle in the IR group compared to controls was significantly increased by DuraGraft^®^ (Figure 1A). In contrast, increased maximum contraction to phenylephrine of aortic rings in the IR group compared to controls was significantly decreased by preservation of aortic rings with DuraGraft^®^ (Table 1, Figure 1B). Sensitivity (pD_2_-value) to phenylephrine was significantly increased in the IR+DuraGraft^®^ rings compared to control and IR groups (Table 1). 

#### 2.1.2. Effect of DuraGraft^®^ on Endothelial Function

To evaluate the effect of DuraGraft^®^ on endothelial function after IRI, aortic rings were precontracted with PE followed by adding increasing concentrations of acetylcholine (ACh, 10^−9^–10^−4^ M). Significantly reduced ACh-induced endothelium-dependent vasorelaxation in the IR group compared to controls was significantly improved by DuraGraft^®^ (Table 1, Figure 1C). Furthermore, decreased aortic sensitivity (pD_2_-value) to ACh seen after IRI was significantly ameliorated by DuraGraft^®^ (Table 1).

#### 2.1.3. Effects of DuraGraft^®^ on Smooth Muscle Relaxation

To evaluate the effect of DuraGraft^®^ on smooth muscle relaxation after IRI, aortic rings were precontracted with phenylephrine followed by adding increasing concentrations of sodium nitroprusside (SNP, 10^−10^–10^−5^ M). Although there was no difference in relaxation to SNP between control and IR groups, the cumulative concentration-response curve to this endothelium-independent agent in the IR+DuraGraft^®^ group was significantly shifted to the right compared to both control and IR rings (Table 1, Figure 1D).

### 2.2. Effects of DuraGraft^®^ on Aortic Intercellular Adhesion Molecule (ICAM)-1, Nitrotyrosine and Platelet Endothelial Cell Adhesion Molecule (PECAM)-1 Immunoreactivity after Vascular IRI

**Figure 2:**
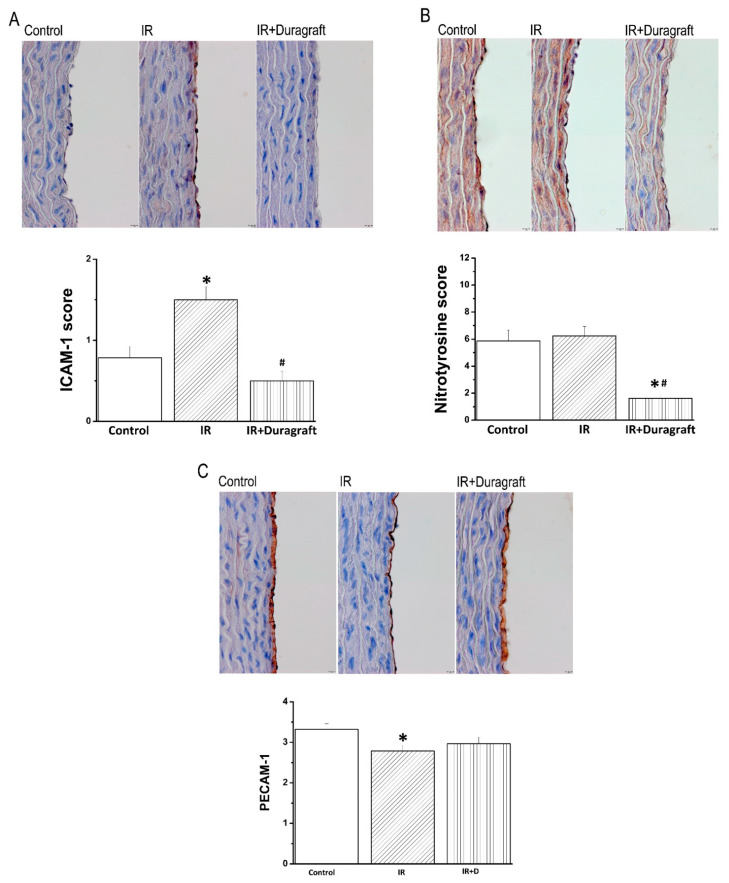
Effect of DuraGraft^®^ on aortic intercellular adhesion molecule (ICAM)-1, nitrotyrosine, and platelet endothelial cell adhesion molecule (PECAM)-1 expression after vascular ischemia/reperfusion (IR) injury. Semi-quantitative scoring of (**A**) ICAM-1, (**B**) nitrotyrosine, and (**C**) PECAM-1 immunohistochemical staining and representative micrographs (×400, scale: 10 µm). Results are represented as mean ± SEM. * *p* < 0.05 versus control; ^#^ *p* < 0.05 versus IR. *n* = 24–28 aortic rings from 6–7 rats/group.

Immunohistochemical analysis for ICAM-1 showed positivity confined to the endothelial layer in the IR group compared to controls, which was significantly decreased by the preservation of aortic rings with DuraGraft^®^ (Figure 2A). Furthermore, DuraGraft^®^ significantly decreased nitrotyrosine immunoreactivity in the IR+DuraGraft^®^ compared to both control and IR groups (Figure 2B). Our results showed that endothelial PECAM-1 immunoreactivity was significantly reduced in the IR rings compared to controls, whereas a similar pattern was observed between control and IR+Duragraft^®^ groups (Figure 2C).

## 3. Discussion

In the present work, we hypothesized that DuraGraft^®^ protects arterial grafts against IRI in a rat model. To the best of our knowledge, this is the first study suggesting that DuraGraft^®^ alleviates both endothelial and contractile dysfunction following in vitro vascular IRI, in part, by lowering inflammatory response through ICAM-1 and decreasing nitro-oxiative stress, without leukocyte engagement. 

CABG is an operation to treat blockages/narrowing of coronary arteries that supply blood to the myocardium, in most cases with an autologous vessel. Intact endothelial cells of the implanted bypass graft is obligatory to prevent smooth muscle cell proliferation and platelet aggregation. Endothelial dysfunction of bypass graft leads to unfavorable complications after bypass surgery (occlusion of late vasculopathy). To preserve endothelial integrity after harvesting, the grafts are temporarily stored in a preservation solution, including physiologic saline, autologous blood and buffered saline. The available preservation methods of the arterial/venous grafts do not sufficiently protect the endothelium and have a detrimental effect on it [9]. In the present study, an isolated tissue bath has allowed ex vivo evaluation of the adverse effects of IRI on vascular function. Our results confirmed that in aortic rings, IRI decreased endothelium-dependent vasorelaxation and sensitivity to exogenous ACh, impaired contractile responses produced by both high KCl-induced depolarization and an α-adrenergic receptor agonist phenylephrine. Among other things, inflammation and oxidative stress play an important role in IR-induced vascular damage [10]. It has been shown that major intercellular adhesion molecule ICAM-1 expression can be enhanced by several inflammatory cytokines [11], as well as by cytokine-independent stimuli, such as ROS, reactive nitrogen species, and hypoxia [12]. In line with these observations, our results demonstrated that IRI significantly increased the protein expression levels of ICAM-1, a master regulator of cellular response in inflammatory processes, nitrotyrosine, a biomarker of nitrosative modification of proteins, and decreased the expression of the endothelial marker PECAM-1. 

In clinical settings, CABG success is limited by graft failure, and the endothelium is the primary target of IRI. The pathophysiology of bypass graft occlusion within the first postoperative month is predominantly thrombosis triggered by surgical trauma and/or endothelial dysfunction caused by IRI [13] An important therapeutic strategy is to preserve and protect endothelial/smooth muscle cell function against IRI. DuraGraft^®^, a novel endothelial-damage inhibitor, has shown to protect the endothelium of vascular conduits during ischemic storage [8]. In accordance with the previous results, we also showed a superior preservation capacity of DuraGraft^®^ over saline solution. DuraGraft^®^ is based on the saline solution; however, it additionally contains glutathione and L-ascorbic acid, L-arginin and other antioxidants. Previous studies have reported powerful endothelium protection by supplementation of the nitric oxide donor L-arginin [14]. Furthermore, human studies demonstrated that DuraGraft^®^ is capable of reducing long-term complications after CABG surgery by preserving the functional and structural integrity of endothelium of the implanted graft [7]. To detect the loss of endothelial cells in the lumen of the grafts, PECAM-1 immunostaining was performed. In the present study, the function and structure of the graft was effectively shown to be preserved using the DuraGraft^®^ solution. Our results additionally demonstrated that DuraGraft^®^ lowered ICAM-1 immunoreactivity, without leukocyte engagement. Furthermore, in the present study, the preservation of IR rings with DuraGraft^®^ decreased nitrotyrosine immunoreactivity, suggesting reduced nitro-oxidative stress. 

This study has certain limitations which have to be pointed out. First, the influence of the surrounding tissues, blood supply, and the activation of leukocytes need to be investigated in an in vivo situation. Second, additional investigations are required to confirm the effects of IRI and DuraGraft^®^ on human internal mammary or radial artery grafts and patients undergoing CABG. Third, an important function of the endothelium as permeability was not directly assessed in the present study. Finally, storage at 4 °C for 24 h is a well-established in vitro model for vascular IRI, even though it does not follow clinical practice. 

## 4. Materials and Methods

### 4.1. Animals

Male Sprague Dawley rats (weighing 250–350 g on arrival, Janvier Labs, Saint Berthevin, France) were housed under a 12–12 h light/dark cycle in a controlled temperature (22 ± 2 °C) room. Food and water were accessible ad libitum and animals were acclimatized for at least for one week before the start of the experiments. All animals received humane care in compliance with the “Principles of Laboratory Animal Care”, formulated by the National Society for Medical Research, and with the “Guide for the Care and Use of Laboratory Animals”, prepared by the Institute of Laboratory Animal Resources and published by the National Institutes of Health (NIH Publication, 8th Edition, 2011) and EU Directive 2010/63/EU. The ethical approval of this study was obtained from the appropriate Institutional Review Board.

### 4.2. Rat Model of Vascular IRI 

#### 4.2.1. Aortic Rings Preparation

The rats were euthanized with an overdose of sodium pentobarbital (120 mg/kg, intraperitoneally). Freshly harvested descending thoracic aorta was placed in a Petri dish filled either with cold (4 °C) oxygenated Krebs–Henseleit solution (KHL) with the following composition (mM): 118 NaCl, 4.7 KCl, 1.2 KH_2_PO_4_, 1.2 MgSO_4_, 1.77 CaCl_2_, 25 NaHCO_3_, and 11.4 glucose, or physiological saline solution. The aorta was then carefully removed from periadventitial fat and connective tissue under magnification before being cutting into 4 mm long pieces.

#### 4.2.2. Aortic Rings Conservation and Experimental Groups

To extrude oxygen, saline or DuraGraft^®^ solutions were gassed with nitrogen. As previously described [15], the aortic rings were placed in test tubes containing 0.9% saline (IR group, *n* = 28–35 rings, 7 rats) or DuraGraft^®^ (IR+DuraGraft^®^ group (*n* = 36 rings, 9 rats), and stored for 24 h at 4 °C. Following cold ischemic conservation, the rings were mounted in organ baths. To mimic free radical generation and endothelial dysfunction, as occur during reperfusion and re-oxygenation in vivo, 200 µM sodium hypochlorite was added to the organ chambers for 30 min. Aortic rings in the control group (*n* = 21–28 rings, 7 rats) were immediately mounted in isolated tissue baths after preparation without cold ischemic storage and hypochlorite incubation.

#### 4.2.3. Ex Vivo Measurement of Vascular Contraction-Relaxation in Organ Baths

Aortic rings were mounted on a stainless-steel hook in organ chambers containing 30 ml of KHL and continuously bubbled with 95% O_2_ and 5% CO_2_ at 37°C and pH 7.4 (EMKA Technologies S.A.S, Paris, France). As previously reported [15], the tissue was initially equilibrated for 20 min at a resting tension before any experimental intervention. During an additional 60 min of equilibration, the passive tension was adjusted periodically to 2 g, during which the baths were rinsed with fresh KHL every 30 min, a precaution against interfering metabolites. At the beginning of each experiment, a pre-contraction was achieved by adding potassium chloride (KCl, 80 mM) to the organ baths to ensure tissue viability and prepare the rings for stable contractions. After the contractile response had stabilized for approximately 30 min, aortic rings were washed until resting tension was again restored. Then, the rings were contracted with an α-adrenergic receptor agonist, phenylephrine (10^−9^–10^−5^ M) until a stable plateau was reached, and the relaxation responses were investigated by adding cumulative concentrations of endothelium-dependent vasorelaxant acetylcholine (ACh, 10^−9^–10^−4^ M). For testing relaxation responses of smooth muscle cells, an endothelium-independent dilator sodium nitroprusside (SNP, 10^−10^–10^−5^ M) was used in phenylephrine (10^−6^ M)-precontracted aortic rings. The contractility of phenylephrine was evaluated as a percentage of the KCl response, and relaxation was expressed as a percentage of the contraction induced by PE. Half-maximal response (EC_50_) to PE, ACh, or SNP were determined from each individual concentration–response curve by sigmoidal fits using Origin 7.0 (MicroCal Software, Northampton, MA, USA). The sensitivity pD_2_ (-logEC_50_) was then calculated.

### 4.3. Immunohistochemical Staining for ICAM-1, Nitrotyrosine and PECAM-1

As previously described [15], immunohistochemistry was performed on buffered paraformaldehyde solution (4%) fixed, paraffin embedded, distal regions of the aortic segments. Four µm thick sections were cut with the Leica microtome (Leica Biosystems Nussloch GmbH, Nussloch, Germany) and placed on slides. Fifteen minutes of hydrogen peroxide (3%) was used to prevent the endogenous peroxidase activity. To unmask the antigenic epitopes, the sections were pre-treated in sodium citrate buffer (pH = 6) for 20 min by heating in a microwave oven at 700 Watt, then blocked in 2% normal serum for 30 min at room temperature. After that, the sections were incubated overnight at 4 °C with mouse monoclonal IgG anti-ICAM-1 (1:100; Abcam, Cambridg, UK), mouse monolyclonal IgG2b anti-nitrotyrosine (1:1000; Abcam, Cambridge, UK) and rabbit monolyclonal IgG anti-PECAM-1 (1:10.000; Abcam, Cambridge, UK) antibodies. The samples were then incubated for 30 min with a biotinylated secondary antibody diluted in serum buffer (1:50), and immunoreactivity was visualized by avidin-biotinylated complex (ABC) reagent (VECTASTAIN universal elite ABC kit, Burlingame, CA, USA). Next, 3,3’ diaminobenzidine (DAB substrate) was added to produce a brown-colored reaction product in the presence of horseradish peroxidase enzyme and used in double labeling applications (VECTOR DAB kit, Burlingame, CA, USA). During the last step, slide sections were cleared before mounting with ProTaqs Mount Aqua (Quartett, Berlin, Germany) and counterstained with haematoxylin. Semi-quantitative immunohistochemical analysis was performed using a conventional light microscope and CellSens software (Olympus Soft Imaging Solutions GmbH, Münster, Germany) based on the distribution patterns score multiplied by area score (0–12). The evaluation of four randomized non-overlapping fields of the aorta was carried out in a blinded fashion. 

### 4.4. Statistical Data Analysis

Data are presented as the mean ± standard error of the mean (SEM). Statistical analyses were performed using GraphPad Prism 7.02 software (GraphPad Software, Inc., San Diego, CA, USA) and graphs were created with Origin 7.0 (MicroCal Software, Northampton, MA, USA). For contractile responses to KCl, pD_2_ values and histological results, the Shapiro–Wilk normality test was used to assess normal distribution. For data with normal distribution, one-way ANOVA followed by Tukey’s post hoc test was carried out for multiple comparisons. If the data were not normally distributed, the nonparametric Kruskal–Wallis test followed by Dunn’s post hoc test was used. The comparison of vascular response curves to PE, ACh and SNP was performed by two-factor mixed ANOVA (factors: DuraGraft^®^ and concentration of reagents (PE, ACh, SNP)) and followed by Tukey’s post hoc test, which was used for multiple comparisons. A *p* value less than 0.05 was considered significant.

## 5. Conclusions

DuraGraft^®^ alleviates vascular dysfunction following in vitro IRI, in part, by reducing nitro-oxidative stress and by lowering inflammatory response through ICAM-1, without leukocytes engagement.

## Figures and Tables

**Table 1:** Quantitative analysis of vascular function after treatment with DuraGraft^®^ against ischemia/reperfusion (IR) injury.

	Control	IR	IR + Duragraft
PE (%) to KCl pD_2_ to PE	80.9 ± 3.2 7.49 ± 0.08	443.4 ± 61.2 * 7.54 ± 0.08	116.3 ± 6.6 *^,#^ 8.05 ± 0.15 *^,#^
KCl (g)	4.2 ± 0.1	1.3 ± 0.2 *	2.6 ± 0.1 *^,#^
R_max_ to ACh (%)	80,2 ± 2.2	47.8 ± 3.0 *	72.9 ± 1.8 ^#^
pD_2_ to ACh	7.39 ± 0.06	6.27 ± 0.20 *	7.05 ± 0.12 ^#^
R_max_ to SNP (%)	100.0 ± 0.0	100.0 ± 0.0	94.0 ± 2.0 *^#^
pD_2_ to SNP	8.99 ± 0.16	8.68 ± 0.09	7.65 ± 0.21 *^#^

Data are represented as mean ± SEM. PE indicates phenylephrine and was expressed as a percentage of the maximum contraction induced by 80 mM potassium chloride (KCl); ACh indicates acetylcholine; SNP, sodium nitroprusside; R_max_, maximum relaxation, and pD_2_, negative logarithm of the corresponding half-maximal response (EC_50_). * *p* < 0.05 versus control; ^#^ *p* < 0.05 versus IR. *n* = 21–36 aortic rings from 7–9 rats/group.

## Data Availability

Data is contained within the article.
